# Involvement of *Trichoderma harzianum* Epl-1 Protein in the Regulation of *Botrytis* Virulence- and Tomato Defense-Related Genes

**DOI:** 10.3389/fpls.2017.00880

**Published:** 2017-05-29

**Authors:** Eriston V. Gomes, Cirano J. Ulhoa, Rosa E. Cardoza, Roberto N. Silva, Santiago Gutiérrez

**Affiliations:** ^1^Department of Biochemistry and Immunology, Ribeirão Preto Medical School, University of São PauloRibeirão Preto, Brazil; ^2^Department of Biochemistry and Cellular Biology, Biological Sciences Institute, Federal University of GoiásGoiânia, Brazil; ^3^Area of Microbiology, University School of Agricultural Engineers, University of LeónPonferrada, Spain

**Keywords:** *Trichoderma harzianum*, Epl-1 protein, *Botrytis cinerea*, fungus–plant interaction, mycoparasitism

## Abstract

Several *Trichoderma* spp. are well known for their ability to: (i) act as important biocontrol agents against phytopathogenic fungi; (ii) function as biofertilizers; (iii) increase the tolerance of plants to biotic and abiotic stresses; and (iv) induce plant defense responses via the production and secretion of elicitor molecules. In this study, we analyzed the gene-regulation effects of *Trichoderma harzianum* Epl-1 protein during the interactions of mutant Δ*epl-1* or wild-type *T. harzianum* strains with: (a) the phytopathogen *Botrytis cinerea* and (b) with tomato plants, on short (24 h hydroponic cultures) and long periods (4-weeks old plants) after *Trichoderma* inoculation. Our results indicate that *T. harzianum* Epl-1 protein affects the *in vitro* expression of *B. cinerea* virulence genes, especially those involved in the botrydial biosynthesis (*BcBOT* genes), during the mycoparasitism interaction. The tomato defense-related genes were also affected, indicating that Epl-1 is involved in the elicitation of the salicylic acid pathway. Moreover, Epl-1 also regulates the priming effect in host tomato plants and contributes to enhance the interaction with the host tomato plant during the early stage of root colonization.

## Introduction

Tomato is one of the most commonly consumed fresh vegetables worldwide, and tomato plants (*Solanum lycopersicum*) are considered as model organisms for studies in different research fields. Tomatoes present a high content of phenolic compounds (Alimohammadi et al., 2017), which have been reported to show preventive and therapeutic effects, working as a signaling molecule and/or interacting with a wide range of molecules in different kinds of diseases, including cancer (Canene-Adams et al., 2005; Blanco-Ulate et al., 2013; Alimohammadi et al., 2017). In addition, tomato plants have been used as informative experimental system in host–pathogen interaction, in particular to necrotrophic fungus *Botrytis cinerea* (Soylu et al., 2010; Malmierca et al., 2012; Blanco-Ulate et al., 2013). Traditional methods used to protect crops from diseases have been based on the use of chemical pesticides. However, application of fungicides and fumigants can have drastic effects on the environment and the consumer’s health. Moreover, these chemicals are often applied in greater quantities than herbicides and insecticides in agricultural practices (Fraser, 1994). These chemical-based methods of crop protection are not an economical solution for the long term because they pollute the atmosphere, damage the environment, leave harmful residues, and can lead to the development of resistant strains among the organisms targeted for control or eradication (Naseby et al., 2000). Nevertheless, alternative strategies have been recently reported, based in the use of essential oils of various plants against the tomato gray mold disease agent *B. cinerea* (Soylu et al., 2010). Furthermore, one of the most promising strategies to reduce, or even eliminate, the chemical treatment of crops is based on the use of biocontrol agents (BCAs), which are reported to have a minimal effect on the environment (Chet and Inbar, 1994; Harman and Kubicek, 1998). Some *Trichoderma* species are known for their biocontrol activity against phytopathogenic fungi (Harman et al., 2004; Lorito et al., 2010; Monteiro et al., 2010). These *Trichoderma* spp. can exert their biocontrol activities through different mechanisms, such as mycoparasitism (Monteiro and Ulhoa, 2006; Almeida et al., 2007), the production of extracellular hydrolytic enzymes (Benítez et al., 2004; Monteiro et al., 2010), the expression of proteins involved in the competition for nutrients which provide a nutritional advantage (Elad, 2000), and the production of secondary metabolites with antifungal activity (Rubio et al., 2009; Cardoza et al., 2011; Tijerino et al., 2011; Malmierca et al., 2012, 2013, 2015). Moreover, some *Trichoderma* spp. strains can interact directly with roots resulting in an increase of plant growth potential, resistance to diseases, and tolerance to abiotic stresses (Shoresh et al., 2010; Hermosa et al., 2012). Proteins from the cerato-platanin (CP) family, such as Sm1/Epl-1 produced by *Trichoderma virens* and *T. atroviride*, can form protein biofilms at air–water interfaces. This feature enhances the polarity effects of surfaces and solutions and thus promotes the formation of highly ordered monolayers at hydrophobic surface–liquid interfaces (Frischmann et al., 2013; Bonazza et al., 2015). The structure of CP proteins resembles that of expansins, which do not have an enzymatic function. Furthermore, expansin-like proteins are important in the degradation of the plant cell wall by inducing the opening of physical spaces required for the action of cellulolytic enzymes (Baccelli et al., 2014; Castro et al., 2014). Like expansins, CP proteins also bind carbohydrates, which suggest that CP proteins have a similar function and that these proteins can induce the opening of physical spaces in structural components of the fungal cell wall, such as in chitin polymers (Frischmann et al., 2013; Baccelli et al., 2014; Bonazza et al., 2015). CP proteins are also effective elicitors capable of triggering local and systemic plant defense responses (Djonovic et al., 2006, 2007; Seidl et al., 2006; Vargas et al., 2008; Pazzagli et al., 2014). In addition, they promote the growth of tomato plants (*Solanum lycopersicum*) by biomass gain, trigger phytoalexin production, and induce cell death in host and non-host plants (Pazzagli et al., 1999; Boddi et al., 2004; Scala et al., 2004; Salas-Marina et al., 2015). The interaction of *T. harzianum* Epl-1 with pathogens and host plants has been previously analyzed, specifically during the mycoparasitism process where Epl-1 plays important roles in: hyphal coils around the fungal host, cell wall protection, hyphal self-recognition, and in the induction of pathogen resistance in bean plants (Gomes et al., 2015). In the present work, and based in those previous results we analyzed the effect of *T. harzianum* Epl-1 on the expression of virulence genes from the phytopathogenic fungus *B. cinerea*, to determine if in addition to regulate the *Trichoderma* hyphal coiling ability around the pathogen, Epl-1 also has some effect on the regulation of *Botrytis* virulence. We also analyzed the importance of Epl-1 on the *Trichoderma*-tomato plant interaction, determining its involvement in the early stages of *Trichoderma* root colonization in hydroponic cultures as well as in the regulation of defense-related genes in 4-weeks old tomato plants.

## Materials and Methods

### Microorganisms and Culture Conditions

*Trichoderma harzianum* strains were provided by the Department of Biochemistry and Molecular Biology, Biochemistry Laboratory, Federal University of Goiás (Goiânia, Brazil), and by the Department of Biochemistry and Immunology, Ribeirão Preto Medical School, University of São Paulo (Ribeirão Preto, Brazil). *T. harzianum* strains were maintained on potato dextrose agar (PDA) medium (Becton Dickinson, Heidelberg, Germany) and sporulated on PPG [2% (dry wt/vol) dehydrated potato flakes (Nestlé, Barcelona, Spain), 2% glucose (Panreac Applichem, Barcelona, Spain), and 2% agar (Becton Dickinson)]. *B. cinerea* B05.10 strain was provided by the Laboratory of Microbiology, University of León (Ponferrada, Spain), and was maintained on malt extract agar (MEA) medium [2% glucose (PA), 2% malt extract (PA), 1% peptone (BD), and 2% agar (BD)], pH 5.6.

### Generation of an *Epl-1* Knockout (Δ*epl-1*) *T. harzianum* Strain

An *epl-1* knockout (Δ*epl-1*) *T. harzianum* strain was generated as described in Gomes et al. (2015). Briefly, for construction of the *epl-1* deletion vector, *epl-1* flanking regions containing 950 bp of the promoter and 1000 bp of terminator regions were amplified using genomic DNA as template, digested with the appropriate restriction enzymes and cloned into a pBluescript SK+ vector (Stratagene, La Jolla, CA, United States), containing a hygromycin B phosphotransferase (*hph*) selection cassette (Mach et al., 1994). Fungal transformation was performed using a protoplasts based procedure as previously described (Gruber et al., 1990; Steiger et al., 2011). The selected transformants were analyzed by PCR and Southern blot hybridization (Gomes et al., 2015).

### Confrontation Assays between *T. harzianum* and *B. cinerea* Strains

**FIGURE 1:**
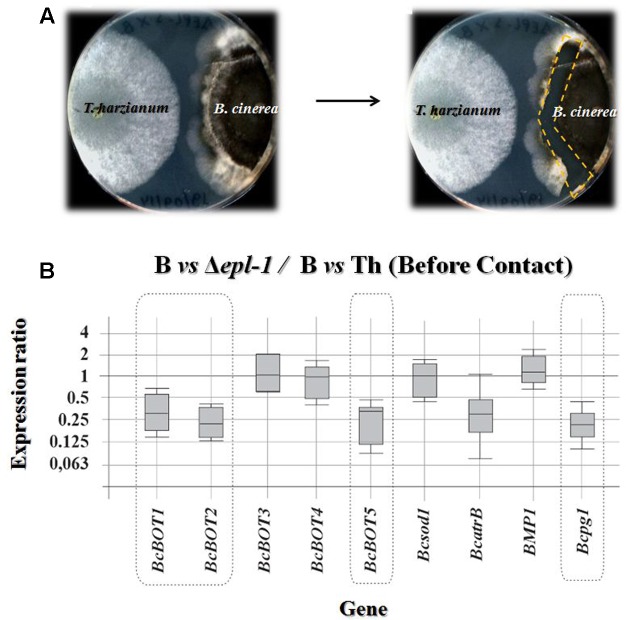
**(A)** Photographs showing confrontation plate assays between *T. harzianum* and *B. cinerea* before hyphae contact. The right panel shows the region from which the *B. cinerea* mycelia was extracted (dashed line). **(B)** qPCR analysis of the relative expression level of several *Botrytis* virulence genes in mycelia confronted with *T. harzianum* Δ*epl-1* (Δ*epl-1*) in comparison with the level of expression in mycelia of the pathogen (B) confronted with *T. harzianum* wild type (Th) strain, in both cases before hyphae contact. The expression ratios as well as the statistic probability values were calculated using the REST2009 software (Pfaffl et al., 2002). Expression ratio values statistically significant [*P(H1)* < 0.05] are outlined by a broken line. Numeric values are included in Supplementary Table S1a. Boxes represent the interquartile range, or the middle 50% of observations. The dotted line represents the median gene expression. Whiskers represent the minimum and maximum observations.

**FIGURE 2:**
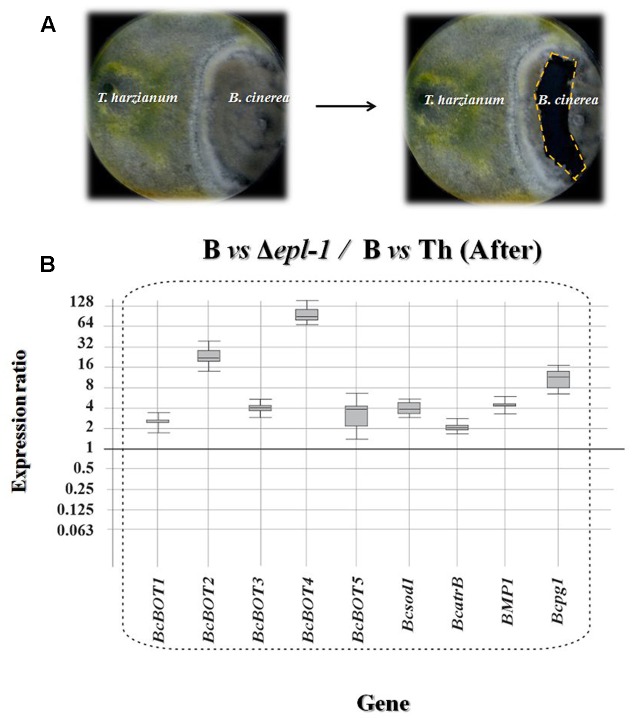
**(A)** Photographs showing confrontation plate assays between *T. harzianum* and *B. cinerea* after hyphae contact. The right panel shows the region from which the *B. cinerea* mycelia was extracted (dashed line). **(B)** qPCR analysis of the relative expression level of several *Botrytis* virulence genes in mycelia confronted with *T. harzianum* Δ*epl-1* (Δ*epl-1*) in comparison with the level of expression in mycelia of the pathogen (B) confronted with *T. harzianum* wild type (Th) strain after hyphae contact. Comparative calculations and graphic representations were carried out as indicated in the legend of **Figure 1**. Numeric values are included in Supplementary Table S1a.

*Trichoderma harzianum* strains were grown for 5 days on PPG at 28°C in the dark and *B. cinerea* B05.10 was grown for 7 days on MEA medium at 21°C with a photoperiod of 16 h light/8 h dark. One agar plug (7 mm in diameter) with growing mycelia of *B. cinerea* was placed on the edge of a 150-mm petri dish containing 50 mL of MEA medium with a sterile cellophane membrane placed on its surface to facilitate the recovery of mycelia from the plate. These plates were incubated at 21°C with a photoperiod of 16 h light/8 h dark. After 4 days of *B. cinerea* growth (about 40 mm in diameter), an agar plug of *T. harzianum* culture was placed on the surface of the plate, opposite to the *B. cinerea* plug. The confrontation plates were incubated at 28°C for 7 days. Both fungi usually began to contact 4 days after the addition of the *Trichoderma* plug. Samples of mycelia were collected from the interaction regions for RNA extraction and analysis by quantitative PCR (qPCR) (**Figures 1A**, **2A**).

### Tomato Plant Assays

*Solanum lycopersicum* var. Marmande tomato seeds were surface sterilized by washing for 10 min in 70% ethanol, followed by 10 min in 50% sodium hypochlorite, and then washed thoroughly in sterile distilled water. Tomato seeds were coated with an aqueous suspension containing 2 × 10^8^ spores of *T. harzianum* per mL (1 mL of spore suspension was used per 30 seeds) and then air dried in an open petri dish overnight under a laminar flow hood. Treated tomato seeds were sown in pots (32 pots per condition) containing commercial loamy field soil [(Kekkilä 50/50); Projar S.A., Valencia, Spain], with the following composition: 90% organic matter (coconut shells and peat), 10% ash, 50% humidity, 1–4/1–6 decomposition degree, pH 5.5 to 6.6, 0.2% nitrogen, 0.1% total phosphorus, 0.5% calcium, 0.1% magnesium, 0.2% iron, 1.5 mg/kg of chlorides, and 80 g/L of dry weight, previously autoclaved at 121°C for 1 h on two consecutive days. Pots with untreated tomato seeds were used as controls. The pots were incubated in a greenhouse at 22 ± 2°C and watered as needed. Four-week-old plants obtained from tomato seeds coated with wild-type or mutant (Δ*epl-1*) *T. harzianum* strains, or not coated (control plants), were infected on the leaf surface with 15 μL of *B. cinerea* conidial suspension, 5 × 10^5^ spores/mL in germination buffer [20 mM glucose (PA) and 20 mM KH_2_PO_4_ (PA)]. Infected plants were incubated for 4 days in a humid chamber under the conditions described above. Two inoculations of *B. cinerea* were made per leaf on 4 leaves per plant for 16 plants per treatment. Leaves were collected for RNA extraction as previously described (Malmierca et al., 2012) from tomato plants before and after infection as well as from non-infected plants.

Tomato-*Trichoderma* hydroponic cultures were carried out as previously described (Rubio et al., 2012). Briefly, sterile stainless-steel screens covered by a sterile gauze sheet, were placed inside Phytatray II boxes (Sigma, St. Louis, MO, United States), and used as a base to support the tomato seeds (30 seeds per box), 1 cm above 100 mL of Murashige and Skoog (MS) medium (Duchefa, Netherlands). Cultures were maintained at 22°C in a plant growth chamber with controlled light and humidity, for 2 weeks. Spores of *T. harzianum* strains (10^7^ spores) were used to inoculate 250-mL flasks containing 100 mL of PDB (BD) medium. Each strain was pre-cultured at 28°C and at 200 rpm in darkness for 48 h. Mycelia were then harvested by filtration, washed with sterile water, and used to inoculate the 2-week-old tomato plants previously placed inside the Phytatray II boxes. Tomato-*Trichoderma* hydroponic cultures were maintained at 22°C and at 80 rpm for 24 h. Finally, *Trichoderma* mycelia attached to the roots were recovered with a direct cold-water jet and used for gene expression analysis by qPCR.

### DNA/RNA Manipulations

All DNA manipulations were performed by standard techniques (Sambrook et al., 1989). Total fungal and plant RNAs were extracted from the mycelia of each sample using TRIzol^®^ RNA kit (Invitrogen Life Technologies, Carlsbad, CA, United States), according to the manufacturer’s instructions. RNA concentrations were determined using a NanoDrop 2000 (Thermo Scientific, Wilmington, DE, United States), and RNA integrity was verified using 1% agarose gel electrophoresis. cDNA was synthesized using 1 μg of total RNA and a reverse transcription system based on the use of an Oligo(dT)15 primer (Promega, Madison, WI, United States). cDNAs were quantified using a NanoDrop 2000 (Thermo Scientific, Wilmington, DE, United States).

### qPCR Assays

qPCR was carried out using a Step One^TM^ system (Applied Biosystems, Foster City, CA, United States). Reactions were performed in a total volume of 20 μL by adding the following components per reaction: 10 μL Power SYBR Green PCR Master Mix (Applied Biosystems), 0.4 μL forward primer (10 μmol⋅L^-1^), 0.4 μL reverse primer (10 μmol⋅L^-1^), 5 μL cDNA and water to a final volume of 20 μL. The relative expression software tool REST 2009© (Pfaffl et al., 2002) was used to calculate the relative expression values and to determine statistical significance between expression ratios. For each primer pair used, a standard curve was generated with 64, 32, 16, 8, 4, and 2 ng of cDNA to determine the PCR amplification efficiency (*E*-value). Each measurement was made in triplicate.

For comparative studies, previously described oligonucleotides specific for *B. cinerea* virulence genes as well as tomato plant defense-related and housekeeping genes were used (Pinedo et al., 2008; Cardoza et al., 2011; Tucci et al., 2011). For tomato plants, gene markers for the salicylic acid (SA) (*PR1b1* and *PR-P2*) and the jasmonate (JA)/ethylene (ET) (*PINI*, *PINII*, and *TomLoxA*) defense-related pathways were analyzed. The pathogenesis-related (PR) protein 1b1 gene (*PR1b1*) is a member of the *PR1* gene family that is used as a marker of SA-mediated responses, whereas PR protein 2 gene (*PR-P2*) is a member of the *PR4* gene family whose expression is regulated by SA. *PINI* and *PINII* are proteinase inhibitor encoding genes that are induced through the JA signal transduction pathway. *TomLoxA* encodes a lipoxygenase enzyme (from the *LOX* family) involved in JA response and defense (Tucci et al., 2011). The expression levels of these genes were compared between the following groups of tomato plants: (1) tomato plants from wild-type *T. harzianum* coated seeds (Tom+Th) versus (vs.) tomato plants from untreated seeds (Tom); (2) tomato plants from *Δepl-1 T. harzianum* coated seeds (Tom+Δ*epl1*) vs. Tom; (3) Tom+Th vs. Tom+Δ*epl1*; and (4) Tom+Th infected with *B. cinerea* (Tom+Th+B) vs. Tom+Δ*epl1* infected with *B. cinerea* (Tom+Δ*epl1*+B). The α*-actin* and *EF1b* genes were used as tomato and *B. cinerea* housekeeping genes, respectively, for gene expression analyses.

### Tomato Root Colonization Index

To quantify the tomato root colonization ability of the *T. harzianum* Δ*epl-1* mutant strain relative to the *T. harzianum* wild-type control strain, oligonucleotides specific for the tomato *GAPDH* and *T. harzianum* α*-actin* genes were used in a qPCR assay. The amount of *Trichoderma* cDNA in the tomato roots was determined by quantifying the level of *Trichoderma* α*-actin* cDNA relative to the level of tomato *GAPDH* cDNA in the same sample. *C*t values were converted to nanograms of cDNA using two calibration curves obtained by plotting the *C*t values of the standards against their known (tomato or *T. harzianum*) cDNA concentrations. Using this approach, root colonization values for the *T. harzianum* Δ*epl-1* mutant strain were calculated relative to the wild-type strain, which was assigned a value of 1. Thus, the values for the Δ*epl-1* mutant were determined by simple mathematical calculations.

## Results

### *T. harzianum* Epl-1 Down-regulates the Expression of *B. cinerea* Virulence Genes after Hyphal Contact

To determine if the expression of *T. harzianum epl-1* affects the expression of genes encoding *B. cinerea* virulence factors, RNA was extracted from mycelia collected at the confrontation areas between *B. cinerea* B05.10 (referred to as B05.10) and wild-type or mutant Δ*epl-1 T. harzianum* before (**Figure 1A**) and after (**Figure 2A**) hyphae contact, and analyzed by qPCR to determine the expression level of representative B05.10 virulence genes. The expression level of the following B05.10 virulence genes was analyzed: five genes involved in the biosynthesis of botrydial (BOT) (*BcBOT5*, *BcBOT3*, *BcBOT2*, *BcBOT1*, and *BcBOT4*), one virulence gene encoding a superoxide dismutase (*Bcsod1*), a gene encoding an ABC transporter (*BcatrB*), as well as a mitogen-activated protein kinase (MAPK) (*BPM1*), and an endopolygalacturonase 1 (*Bcpg1*) encoding genes. Our results showed that, before hyphal contact, the expression ratios (shown as fold-changes) of *BcBOT1*, *BcBOT2*, *BcBOT5*, and *Bcpg1* were slightly down-regulated in the confrontation with Δ*epl-1* mutant (**Figure 1B**). In addition, the expression of most B05.10 virulence genes analyzed was not affected before hyphal contact by the interaction with the wild-type *T. harzianum* strain, except for two genes, *BcatrB* and *Bcpg1*, which were slightly up-regulated as compared to their respective levels in B05.10 hyphae growing alone (control condition, B) (Supplementary Figure S1A and Table S1b).

After hyphal contact, all B05.10 virulence genes analyzed were up-regulated in *T. harzianum* Δ*epl-1* confrontation areas, compared to their respective expression levels in wild-type *T. harzianum* confrontation areas (**Figure 2B**), suggesting that the lack of Epl-1 resulted in a higher level of expression of *B. cinerea* virulence genes and therefore Epl-1 would be involved in the overall reduction in virulence activity of the pathogen. Furthermore, after hyphal contact with wild-type *T. harzianum*, six B05.10 genes were significantly down-regulated (*BcBOT1*, *BcBOT2*, *BcBOT4*, *BcBOT5*, *BcatrB* and *BPM1*) and two genes (*Bcsod1* and *Bcpg1*) were up-regulated when compared to their respective levels in B05.10 hyphae growing alone (Supplementary Figure S2A and Table S1b). In contrast, in *T. harzianum* Δ*epl-1* confrontation areas six B05.10 virulence genes (*BcBOT3*, *BcBOT4*, *Bcsod1*, *BPM1* and *Bcpg1*) were up-regulated and only *BcatrB* was strongly down-regulated, when compared with the level of expression in B05.10 growing alone (Supplementary Figure S2B and Table S1b). These results indicate that the mutant *T. harzianum* Δ*epl-1* has lost its ability to down-regulate the expression of *B. cinerea* virulence genes and suggest that Epl-1 plays an important role in *T. harzianum*-mediated biocontrol of this phytopathogen fungus.

### *T. harzianum* Epl-1 Protein Up-regulates the Expression of *PR-P2*, a Gene Involved in SA-Mediated Response

**FIGURE 3:**
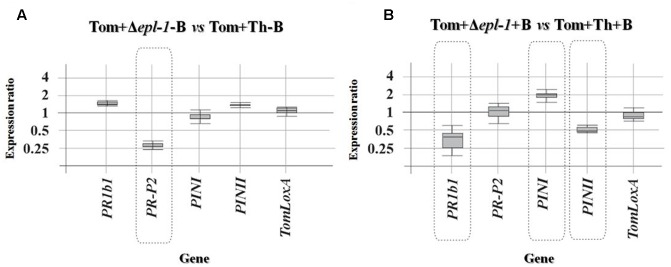
**Relative expression ratios of defense-related genes belonging to the SA and JA defense-related pathways in 4-week-old tomato plants (Tom) inoculated with the *T. harzianum* Δ*epl-1* mutant (Δ*epl-1*) versus plants inoculated with *T. harzianum* wild-type strain (Th). (A)** Plants not infected with the pathogen *B. cinerea* B05.10 (–B). **(B)** Plants infected with the pathogen *B. cinerea* B05.10 (+B). Comparative calculations and graphic representations were carried out as indicated in the legend of **Figure 1**. Numeric values are included in Supplementary Table S2A.

We analyzed the expression levels of five tomato marker genes involved in the SA- (*PR1b1* and *PR-P2*) or JA/ET- (*PINI*, *PINII*, and *TomLoxA*) mediated signaling pathways, in leaves collected from 4-week-old tomato plants treated with wild-type or Δ*epl-1 T. harzianum* strains and infected or not with *B. cinerea*. When gene expression levels were compared between non-infected (-B) plants the expression of *PR-P2* was down-regulated in the absence of Epl-1 (**Figure 3A**). Nevertheless, when the same comparison was analyzed in plants infected with the pathogen (+B), no effect was observed on *PR-P2*, while *PR1b1* and *PINII* were down-regulated (**Figure 3B**), and *PINI* was slightly up-regulated. Furthermore, uninfected plants (-B) treated with wild-type *T. harzianum* showed a slight but significant up-regulation of *PR-P2* and a strong down-regulation of *TomLoxA* when compared to control plants (uninfected and not coated with *T. harzianum*) (Supplementary Figure S3A and Table S2b). Similar results were observed when the effect of *T. harzianum* Δ*epl-1* treatment was compared with control plants, with the exception of *PR-P2*, which was significantly down-regulated in this latter case (Supplementary Figure S3B and Table S2b). Thus, these data suggest that the lack of Epl-1 protein resulted in a lower level of expression of genes related to SA response, both in the absence (*PR-P2*) or in the presence (*PR1b1*) of the pathogen.

### *PR-P2* Is also Down-regulated in Tomato Hydroponic Cultures Inoculated with Δ*epl-1* Mutant

**FIGURE 4:**
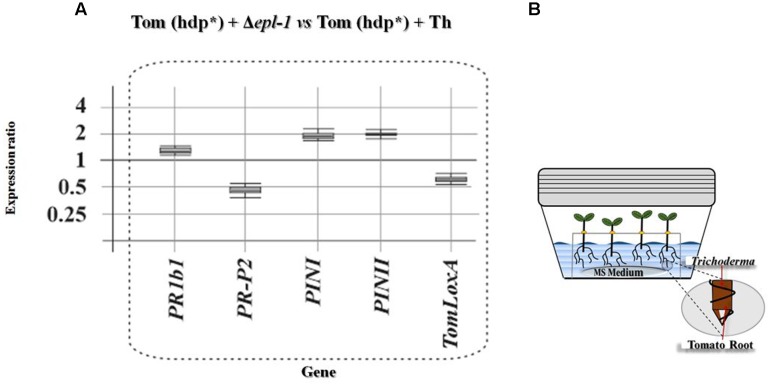
**Relative expression ratios of tomato defense-related genes in hydroponic cultures.** **(A)** Relative expression levels of defense-related genes belonging to the SA and JA pathways in tomato (Tom) hydroponic cultures (hdp^∗^) inoculated with *T. harzianum* Δ*epl-1* (Δ*epl-1*) versus those inoculated with the *T. harzianum* wild-type (Th) strains. **(B)** Illustrative scheme of the hydroponic culture box of tomato plants inoculated with *T. harzianum* strains. Comparative calculations and graphic representations were carried out as indicated in the legend of **Figure 1**. Numeric values are included in Supplementary Table S3a.

In order to confirm the data on the expression of tomato defense-related genes described above, the expression levels of these genes were also determined in 2-week-old tomato plants grown in hydroponic cultures. Incubation of the hydroponic cultures with *T. harzianum* strains for 24 h, significantly affected the expression of all genes belonging to both, the SA, and JA/ET defense-related pathways being analyzed. Comparison of the gene expression levels in plants treated with the Δ*epl-1* mutant or with the wild-type *T. harzianum* strain showed that inoculation with the Δ*epl-1* mutant up-regulated *PR1b1* (SA pathway) as well as *PINI* and *PINII* (JA pathway), and slightly down-regulated *PR-P2* (SA) and *TomLoxA* (JA) (**Figure 4**). When the effect of both *T. harzianum* strains was analyzed independently, *PR1b1* was up-regulated by both strains, but at a higher level by the Δ*epl-1* mutant and *PR-P2* was slightly up-regulated by the wild-type strain but down-regulated by the mutant Δ*epl-1* strain. In the case of the JA/ET pathway-related genes, *PINI* was up-regulated and *TomLoxA* was down-regulated, each by approximately twofold in plants inoculated with the mutant *T. harzianum* Δ*epl-1* strain compared to those inoculated with the wild-type strain (Supplementary Figures S4A,B and Table S3b).

### *T. harzianum* Epl-1 Is Involved in the Early Stages of Tomato Root Colonization

**FIGURE 5:**
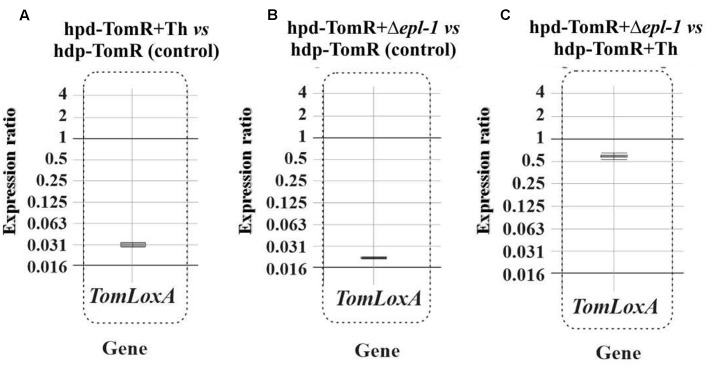
**qPCR analysis of *TomLoxA* expression in hydroponic tomato roots (hdp-TomR) 24 h after *T. harzianum* strains inoculation as a reference gene for tomato roots colonization in:** **(A)** Tomato plants inoculated with *T. harzianum* wild-type (Th) strain versus non-inoculated plants. **(B)** Tomato plants inoculated with *T. harzianum* Δ*epl-1* strain versus non-inoculated plants. **(C)** Fold change of relative gene expression of Tomato plants inoculated with *T. harzianum* wild-type versus expression in Tomato plants inoculated with Δ*epl-1* mutant. qPCR comparative calculations and graphic representations were carried out as indicated in the legend of **Figure 1**. Numeric values are included in Supplementary Table S4a.

Roots from tomato plants grown in hydroponic cultures were collected 24 h after *T. harzianum* inoculation and RNA was extracted for gene expression analysis by qPCR. The expression ratio of tomato *GAPDH* gene relative to the expression of *T. harzianum* α*-actin* gene were used to calculate the index of colonization that was 1.00 and 0.41 for wild-type and Δ*epl-1* inoculated plants, respectively, indicating a lower root colonization by the Δ*epl-1* mutant. These results suggest that Epl-1 is involved in the early stages of *T. harzianum* root colonization, and that disruption of *epl-1* reduces its colonization ability. The analysis of *TomLoxA* (a gene related in the control of the spread of beneficial fungi in roots) relative expression levels showed that this gene is down-regulated in plants inoculated with *T. harzianum* Δ*epl-1* mutant compared with those inoculated with wild-type strain (**Figure 5**), further supporting the root colonization data, and confirming that the lack of Epl-1 affects the ability of *T. harzianum* to colonize tomato roots.

## Discussion

*Trichoderma* spp. are among the most frequently isolated soil fungi present in plant root ecosystems. Many species belonging to this genus are opportunistic, avirulent plant symbionts that act as parasites or antagonists of important phytopathogenic fungi (Harman et al., 2004). Competition for carbon, nitrogen, and space, can also explain how *Trichoderma* spp. controls plant pathogens. Furthermore, *T. harzianum* is able to control phytopathogens, like *B. cinerea*, by colonizing blossom tissues and excluding the pathogen from its infection site (Gullino, 1992; Vinale et al., 2008). Epl-1, a member of the CP protein family, is a well-known *Trichoderma* spp. virulence factor that induces local and systemic defenses in plants, thus playing an important role in the interaction of different organisms with host plants. As mentioned earlier, this protein is essential in several steps of the mycoparasitic process, such as the hyphal coiling that occurs during the interaction with phytopathogenic fungi, e.g., *Sclerotinia sclerotiorum*. In addition, the accumulation of Epl-1 in the *T. harzianum* cell wall contributes to the self-protection of hyphae against degradation by lytic enzymes, as well as to the self- and host-fungal hyphae recognition (Gomes et al., 2015). In the present work, we analyzed the involvement of *T. harzianum* Epl-1 during the interaction with the pathogen *B. cinerea* and with tomato plants. To this end, we evaluated the effects of Epl-1 on the expression of *B. cinerea* virulence genes and on plant defense-related genes, as well as its role in root colonization by *Trichoderma*.

Multiple sesquiterpene biosynthetic gene clusters have been described in filamentous fungi, such as those involved in the biosynthesis of trichothecenes by *Fusarium*, *Myrothecium*, and *Trichoderma* spp. (Desjardins et al., 1993; Trapp et al., 1998; Cardoza et al., 2011; McCormick et al., 2011), and botrydial by *B. cinerea*. BOT genes are one of the most important virulence factors of *Botrytis* spp., and their expression during plant infection induces chlorosis and cell collapse (Deighton et al., 2001; Pinedo et al., 2008). Furthermore, BOT has been reported as a repressor of the gene cluster responsible for the biosynthesis of harzianum A (HA), a trichothecene produced by *T. arundinaceum* that lacks *in vivo* phytotoxicity. HA is involved in biocontrol activity and promotes the expression of plant defense-related genes (Malmierca et al., 2012, 2016). In this present work, we analyzed the role that Epl-1 has in the modulation of the BOT biosynthetic gene cluster expression in *B. cinerea* during direct confrontation assays and identified Epl-1 as an important regulator of BOT biosynthesis.

Before hyphal contact occurred, the *T. harzianum* Δ*epl-1* mutant unexpectedly down-regulated five *B. cinerea* virulence-related genes whereas most virulence genes analyzed remained unaffected in confrontation assays with *T. harzianum* wild-type (**Figure 1B** and Supplementary Figure S1). However, upon fungal hyphal contact, the gene expression pattern changed drastically. Thus, after fungal hyphal contact had occurred, confrontation with the Δ*epl-1* mutant resulted in a strong up-regulation of all analyzed genes (**Figure 2B**), whereas confrontation with the wild-type *T. harzianum* resulted in the down-regulation of six *B. cinerea* virulence genes, with the strongest down-regulation observed for *BcatrB*, which encodes an ABC transporter that supports the growth and the pathogenic activity of *B. cinerea* (Del Sorbo et al., 2008) (Supplementary Figure S2A). These results suggest that Epl-1 is an important factor controlling the expression of *Botrytis* spp. virulence genes. Furthermore, in the absence of Epl-1, a higher degradation of the *Trichoderma* spp. cell wall would be expected resulting in the release of small elicitor molecules that can upregulate *B. cinerea* virulence-related genes (Malmierca et al., 2016). Thus, demonstrating the important function of Epl-1 during the mycoparasitism process (Gomes et al., 2015).

Plants are in a continuous evolutionary battle against a multitude of microbes and other organisms. Pathogens usually access the plant tissues either by penetrating the leaf and root surfaces directly, or by entering through wounds and natural openings such as the leaf stomata. *Trichoderma* spp. colonization triggers a wide array of plant responses, which may result in the enhancement of the plant’s defense strength (Bailey et al., 2006; Marra et al., 2006; Alfano et al., 2007; Morán-Diez et al., 2012; Malmierca et al., 2014). Often, the effects of *Trichoderma* spp. colonization on the plant’s defense system are not restricted solely to root tissues, but may also be present in above-ground tissues (Martínez-Medina et al., 2010, 2011; Salas-Marina et al., 2011; Mathys et al., 2012), which renders the plant resistant to a broad-spectrum of plant pathogens. This systemic resistance is likely the result of the modulation of the plant’s defense signaling network that translates the *Trichoderma*-induced early signaling events into a more efficient activation of defense responses. It is well known that the phytohormones jasmonic acid (JA), SA, and abscisic acid (ABA) act as dominant primary signals on the regulation of local and systemic defense responses in plants (Pieterse et al., 2009), and as a result, play a central role in the phenomena of induced resistance. Generally, fungal biotrophic pathogens induce systemic acquired resistance (SAR), which is dependent on the SA-regulated signaling pathway. In contrast, the induction of systemic resistance (ISR) usually relies on JA signaling and is a consequence of the interaction of the plant with symbiont or necrotrophic microorganisms (Pieterse et al., 1996; van Loon et al., 1998; Durrant and Dong, 2004; Pozo et al., 2008; Van Wees et al., 2008; Van der Ent et al., 2009; Zeilinger et al., 2016). In this report, 4-week-old tomato plants colonized with either wild-type or mutant Δ*epl-1 T. harzianum* strains were analyzed for the expression of marker genes involved in the induction of the SA and JA/ET pathways. Several reports have shown that, in plants challenged with *B. cinerea*, *Trichoderma* spp. colonization induces a transient increase in expression of defense-related genes and the production of secondary metabolites with antimicrobial activity (Yedidia et al., 1999; Yedidia and Shoresh, 2003; Shoresh et al., 2005). However, another report has shown that rhizosphere colonization by *Trichoderma* spp. supports the transcription of some defense-related genes at low, but significant levels, for a relatively long period of time (Alfano et al., 2007; Tucci et al., 2011). This observation is in agreement with our results that identify Epl-1 is an eliciting molecule that can remain activated for a long period, as was demonstrated by altered expression profiles of genes involved in the SA defense-related pathway in plants inoculated with the mutant Δ*epl-1* versus the expression level in those inoculated with wild-type *T. harzianum* strain, prior to pathogen challenge (**Figure 3A**). Moreover, when the plants were challenged with *B. cinerea*, the fold-change in gene expression observed were indicative of a typical plant defense-related pathway induced by an interaction with plant-necrotrophic fungi (**Figure 3B**). A similar relative induction of the SA pathway prior to *B. cinerea* challenge has been previously reported (Tucci et al., 2011; Cardoza et al., 2014; Harel et al., 2014), and this induction may be conditioned by the presence of Epl-1 (Supplementary Figure S3B).

Plant roots colonized with *Trichoderma* spp. have been reported as “sensitized” because they respond faster and/or more intensely to pathogen attack via a mechanism known as “priming effect” (Conrath et al., 2006; Ahn et al., 2007). This priming of defense pathways, upon infection with *B. cinerea*, has been previously reported (Ahn et al., 2007; Tucci et al., 2011; Malmierca et al., 2012; Palmieri et al., 2012; Harel et al., 2014). Martínez-Medina et al. (2016), hypothesized that this effect could be a plastic phenomenon with temporal regulation of the tomato plant infection. The authors argued that *Trichoderma* spp. primes SA- and JA-dependent defenses in tomato roots, and that the levels of priming upon attack with the nematode *Meloidogyne incognita* depend on the parasitism stage. Our results show that Epl-1 elicits the expression of plant defense-related genes at early time points after *Trichoderma* inoculation, suggesting that Epl-1 is directly involved in the priming effect of tomato plants. When tomato hydroponic cultures were analyzed 24 h after *T. harzianum* wild-type inoculation, consistent up-regulation of genes involved in both SA- and JA-defense pathways was observed (Supplementary Figure S4A). A remarkably strong up-regulation of SA- and JA-defense-related genes in plants inoculated with the mutant *T. harzianum* Δ*epl-1* was observed, in particular for *PR1b1*, *PINI*, and *PINII* (Supplementary Figure S4B), which showed relative gene expression levels higher than those induced by *B. cinerea* (data not shown). Another important difference observed between plants inoculated with *T. harzianum* Δ*epl-1* and wild-type strains was the relative down-regulation of *TomLoxA* expression in the roots of tomato hydroponic cultures inoculated with the Δ*epl-1* mutant strain compared to those inoculated with the wild-type strain (**Figure 5**). *TomloxA* has been associated with the spread of beneficial fungi in plant roots (León-Morcillo et al., 2012; Cardoza et al., 2014; Harel et al., 2014). These previous observations agree with the lower colonization ratio observed for the Δ*epl-1* mutant compared to the wild-type strain, indicating that the ability of the mutant to colonize tomato roots was reduced by 59% 24 h after inoculation relative to the colonization rate of the wild-type strain, and that Epl-1 plays a role in the earlier stages of plant–*Trichoderma* interactions during root colonization. The expression values reported here, together with previous data (Gomes et al., 2015) suggest that, the absence of Epl-1 in the *T. harzianum* cell wall, during its interaction with the host plant, would lead to the enzymatic degradation of the cell wall and the release of fungal elicitors. These released fungal elicitors could trigger in turn a signaling cross-talk that results in plant defense responses similar to those observed during plant–phytopathogen interactions. This idea is supported by the observed decrease in root colonization potential during the early stages of *T. harzianum* Δ*epl-1* and tomato root interaction.

## Conclusion

*Trichoderma harzianum* Epl-1 would be a fundamental player in the *Trichoderma*–plant–pathogen interaction, responsible of several processes, including mycoparasitic success, reduction of virulence of pathogenic fungi, and symbiotic association with plants, in which *Trichoderma* spp. induce a priming effect allowing the plant to readily respond against pathogens through multiple mechanisms that depend on the expression of Epl-1. All these aspects would contribute to the evolutionary success of *Trichoderma* spp.

## Author Contributions

EV performed the experimental design, laboratory experiments, bioinformatics analysis, and drafted the manuscript. CU and RC interpreted the data and revised the manuscript. RS and SG designed the project, supervised the research study, interpreted the data, and drafted and revised the manuscript. All the authors have read and approved the final manuscript.

## Conflict of Interest Statement

The authors declare that the research was conducted in the absence of any commercial or financial relationships that could be construed as a potential conflict of interest.
