# Supplementary material for: Involvement of Trichoderma harzianum Epl-1 Protein in the Regulation of Botrytis Virulence- and Tomato Defense-Related Genes
Source: Front Plant Sci. 2017 May 29;8:880. doi: 10.3389/fpls.2017.00880 (PMC5446994; doi:10.3389/fpls.2017.00880)
Supplement: Supplementary file 1 [file Table_1.PDF]

Table S1a. Numeric values of the expression ratios observed for the analyzed genes

	BcBOT1	*P(H1)*	*BcBOT2*	*P(H1)*	*BcBOT3*	*P(H1)*	*BcBOT4*	*P(H1)*	*BcBOT5*	*P(H1)*	*bcsod1*	*P(H1)*	*BcatrB*	*P(H1)*	*BMP1*	*P(H1)*	*Bcpg1*	*P(H1)*
B *vs* Δ*epl-1* / B vs Th (Before Contact)	0.294*	0.000	0.211*	0.048	1.089	0.575	0.847	0.737	0.218*	0.000	0.902	0.734	0.265	0.095	1.208	0.575	0.193*	0.000
B *vs* Δ*epl-1* / B vs Th (After Contact)	2.453*	0.023	21.498*	0.000	3.872*	0.000	87.090*	0.038	3.200*	0.000	3.811*	0.000	2.070*	0.000	4.281*	0.000	10.359*	0.000

Table S1b. Numeric values of the expression ratios observed for the analyzed genes

	BcBOT1	*P(H1)*	*BcBOT2*	*P(H1)*	*BcBOT3*	*P(H1)*	*BcBOT4*	*P(H1)*	*BcBOT5*	*P(H1)*	*bcsod1*	*P(H1)*	*BcatrB*	*P(H1)*	*BMP1*	*P(H1)*	*Bcpg1*	*P(H1)*
B *vs* Th / B (Before contact)	1.027	0.818	2.024	0.162	1.025	0.906	0.750	0.441	1.784	0.229	1.750	0.101	1.933*	0.030	0.920	0.906	2.448*	0.000
B *vs* Δ*epl-1* / B (Before Contact)	0.302*	0.000	0.426*	0.000	1.116	0.324	0.636*	0.031	0.390*	0.000	1.578*	0.010	0.512	0.330	1.112	0.521	0.473*	0.000
B *vs* Th / B (After contact)	0.605*	0.036	0.076*	0.000	0.705	0.103	0.065*	0.026	0.318*	0.000	2.227*	0.037	0.038*	0.000	0.506*	0.000	3.606*	0.000
B *vs* Δ*epl-1* / B (After contact)	1.484	0.131	1.643	0.150	2.731*	0.000	5.641*	0.000	1.017	0.892	8.486*	0.000	0.079*	0.000	2.165*	0.000	37.354*	0.000

Table S2a. Numeric values of the expression ratios observed for the analyzed genes

	PR1b1	*P(H1)*	*PR-P2*	*P(H1)*	*PINI*	*P(H1)*	*PINII*	*P(H1)*	*TomLoxA*	*P(H1)*
Tom+Δ*epl-1* -B *vs* Tom+Th-B	1.451	0.108	0.252*	0.000	0.816	0.092	1.361	0.108	1.085	0.328
Tom+Δ*epl-1* +B *vs* Tom+Th+B	0.368*	0.047	1.041	0.821	1.991*	0.031	0.528*	0.022	0.926	0.469

Table S2b. Numeric values of the expression ratios observed for the analyzed genes

	PR1b1	*P(H1)*	*PR-P2*	*P(H1)*	*PINI*	*P(H1)*	*PINII*	*P(H1)*	*TomLoxA*	*P(H1)*
Tom+Th-B *vs* Tom-Th-B	1.249	0.102	1.491*	0.000	0.759	0.092	0.916	0.236	0.237*	0.000
Tom+Δ*epl-1* -B *vs* Tom-Δ*epl-1* -B	1.812	0.093	0.376*	0.027	0.619*	0.033	1.246*	0.026	0.257*	0.046

Table S3a. Numeric values of the expression ratios observed for the analyzed genes

	PR1b1	*P(H1)*	*PR-P2*	*P(H1)*	*PINI*	*P(H1)*	*PINII*	*P(H1)*	*TomLoxA*	*P(H1)*
Tom (hdp*)+Δ*epl-1* *vs* Tom (hdp*)+Th	1.268*	0.035	0.436*	0.015	1.923*	0.000	1.991*	0.000	0.590*	0.040

Table S3b. Numeric values of the expression ratios observed for the analyzed genes

	PR1b1	*P(H1)*	*PR-P2*	*P(H1)*	*PINI*	*P(H1)*	*PINII*	*P(H1)*	*TomLoxA*	*P(H1)*
Tom (hdp*)+Th vs Tom (hdp*)	42.908*	0.000	1.385*	0.000	82.951*	0.000	3.820*	0.034	0.757*	0.019
Tom (hdp*)+Δ*epl-1* *vs* Tom (hdp*)	54.398*	0.023	0.604*	0.000	159.512*	0.031	7.607	0.098	0.447*	0.000

Table S4a. Numeric values of the expression ratios observed for the analyzed genes

	TomLoxA	*P(H1)*
hdp-TomR+Th *vs* hdp-TomR (control)	0.031*	0.000
hdp-TomR+Δ*epl-1* *vs* hdp-TomR (control)	0.018*	0.000
hdp-TomR+Th *vs* hdp-TomR+Δ*epl-1*	0.585*	0.029

Expression ratio values indicated with an asterisc are statistically significant [*P(H1)* <0.05].

B = *B. cinerea* B05.10; Th = *T. harzianum* wild type; Δ*epl-1* = *T. harzianum* Δ*epl-1* ; Tom = Tomato plants; TomR = Tomato roots; hdp*= hydroponic culture
