# Supplementary material for: Involvement of Trichoderma harzianum Epl-1 Protein in the Regulation of Botrytis Virulence- and Tomato Defense-Related Genes
Source: Front Plant Sci. 2017 May 29;8:880. doi: 10.3389/fpls.2017.00880 (PMC5446994; doi:10.3389/fpls.2017.00880)
Supplement: Supplementary file 2 [file Image_1.PDF]

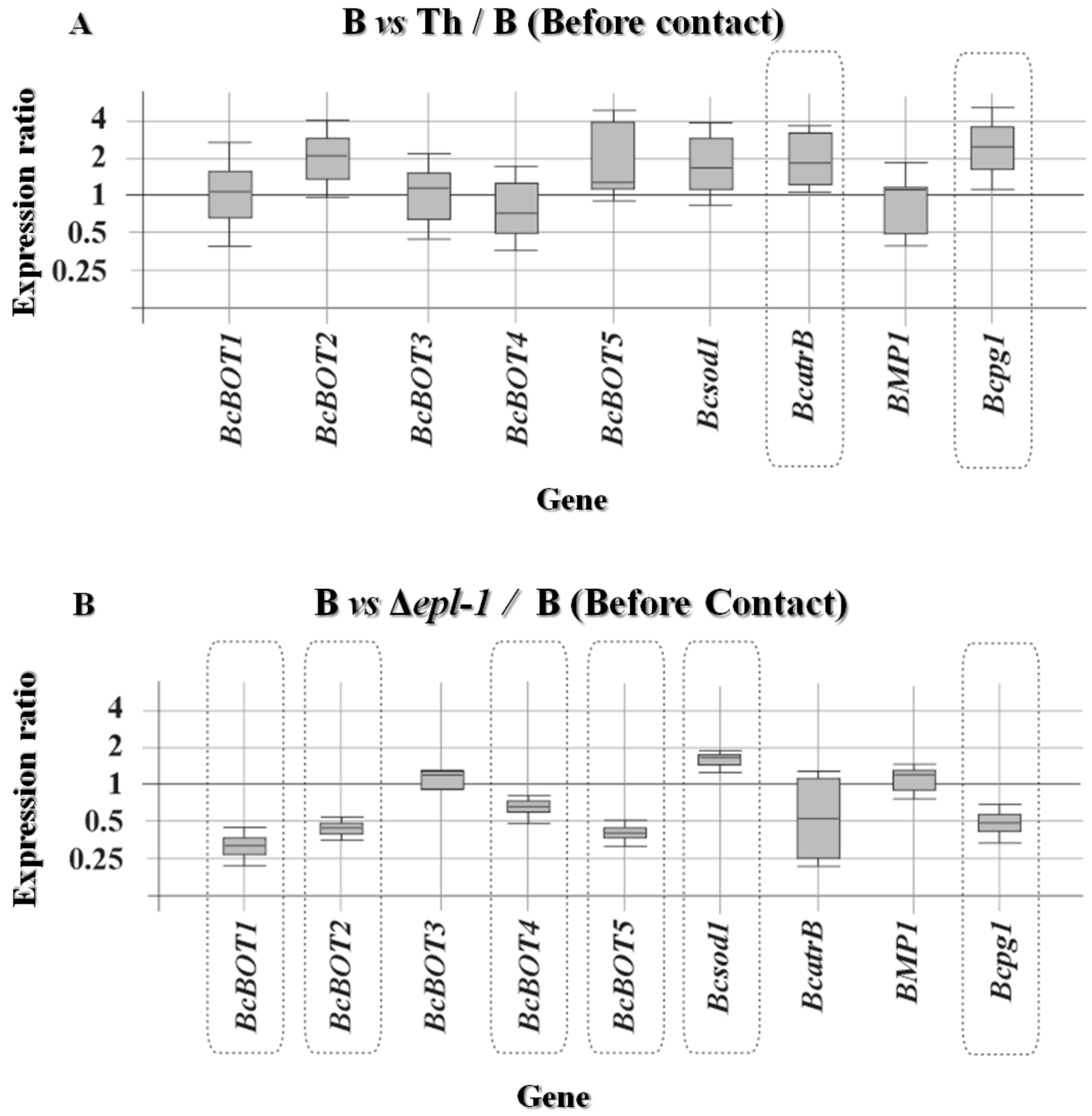

Supplementary Figure S1 – qPCR analysis of the relative expression level of nine *Botrytis* virulence genes in mycelia confronted with *T. harzianum* strains before hyphae contact. **A.** control condition (B) *versus T. harzianum* WT (Th *vs* B). **B.** control condition (B) *versus T. harzianum* Δ*epl-1* (Δ*epl1* vs B). In both cases the comparison was carried out against the values of gene expression in *Botrytis* grown alone. Numeric data are in the Supplementary Table S1b and statistically significant values ($P<0.05$) are indicated with an asterisk (Table S1b) and outlined by a broken line in the graphical representation. *p(H1)* - Probability of alternate hypothesis that difference between sample and control groups is due only to chance. Boxes represent the interquartile range, or the middle 50% of observations. The dotted line represents the median of the gene expression level. Whiskers represent the minimum and maximum observations. The expression ratios as well as the statistic probability values were calculated using the REST©2009 software (Pfaffl et al., 2002).

Pfaffl, M. W. (2002). Relative expression software tool (REST(C)) for group-wise comparison and statistical analysis of relative expression results in real-time PCR. *Nucleic Acids Res.* 30, 36e–36. doi:10.1093/nar/30.9.e36.
