# Supplementary material for: Involvement of Trichoderma harzianum Epl-1 Protein in the Regulation of Botrytis Virulence- and Tomato Defense-Related Genes
Source: Front Plant Sci. 2017 May 29;8:880. doi: 10.3389/fpls.2017.00880 (PMC5446994; doi:10.3389/fpls.2017.00880)
Supplement: Supplementary file 3 [file Image_2.PDF]

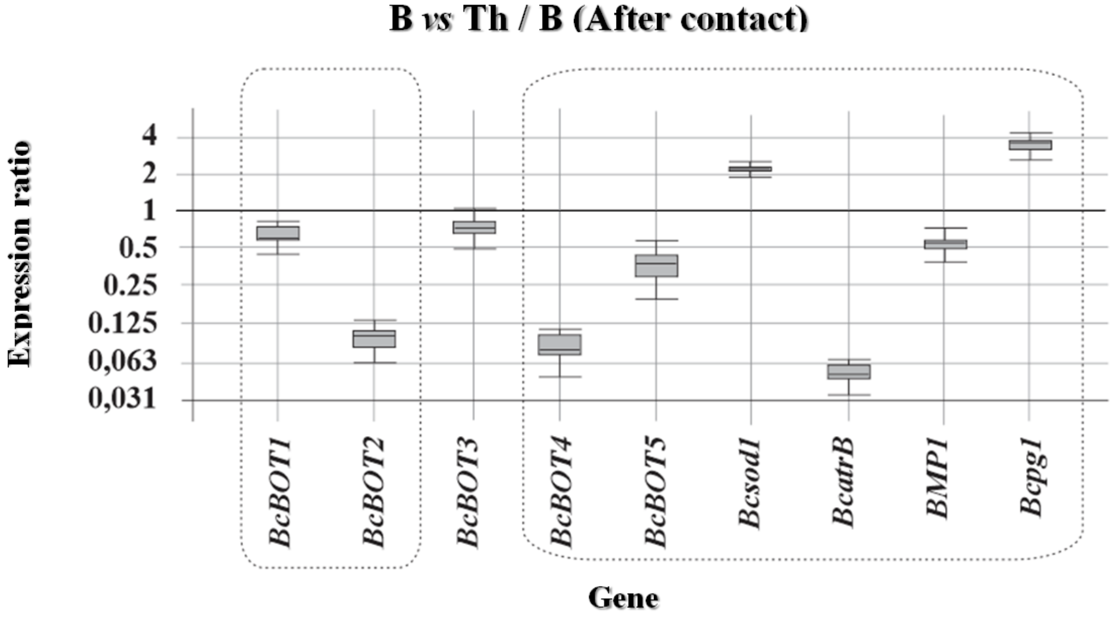

B

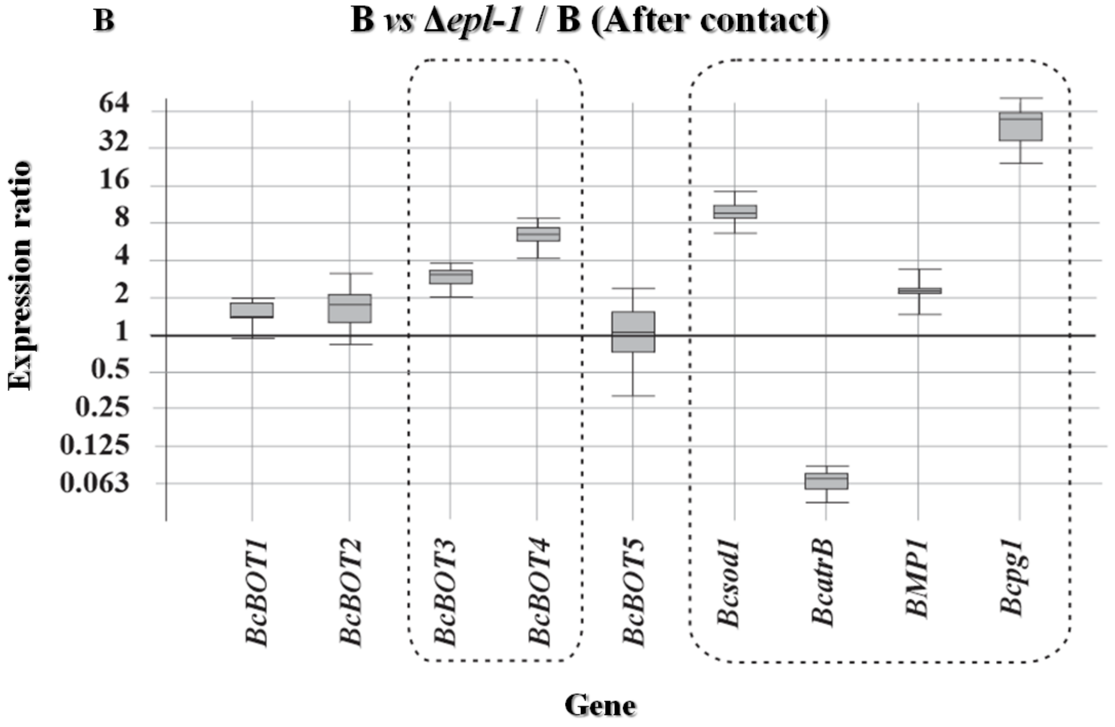

Supplementary Figure S2 - qPCR analysis of the relative expression level of several *Botrytis* virulence genes in mycelia confronted with *T. harzianum* strains after hyphae contact. **A** – B05.10 confronted against *T. harzianum* wild type compared to B05.10 growing alone (*Th vs B / B*). **B** - B05.10 confronted against *T. harzianum* Δ*epl-1* compared to B05.10 growing alone (Δ*epl-1 vs* B / B). qPCR comparative calculations and representations were carried out as indicated in the legend of Supplementary Fig. 1. Numeric values are included in Supplementary Table S1b.
