# Supplementary material for: Involvement of Trichoderma harzianum Epl-1 Protein in the Regulation of Botrytis Virulence- and Tomato Defense-Related Genes
Source: Front Plant Sci. 2017 May 29;8:880. doi: 10.3389/fpls.2017.00880 (PMC5446994; doi:10.3389/fpls.2017.00880)
Supplement: Supplementary file 4 [file Image_3.PDF]

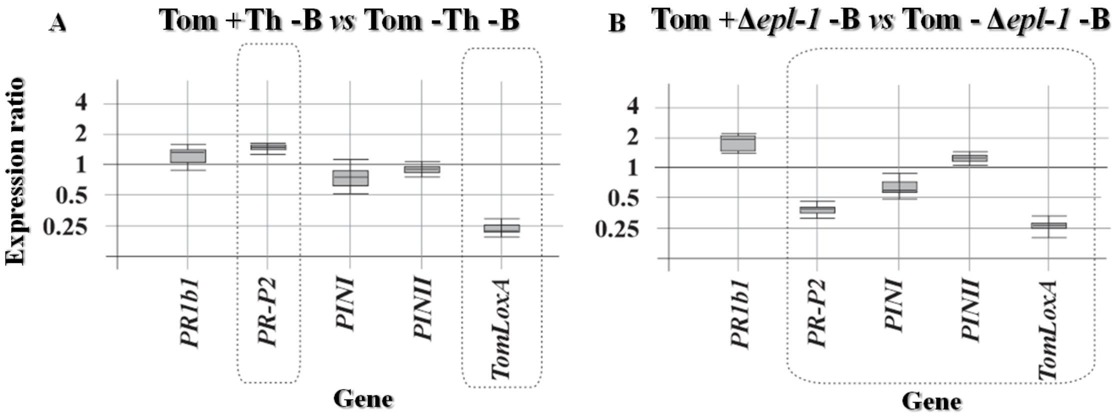

Supplementary Figure S3 – Relative expression levels of genes belonging to the SA and JA defense-related pathways in 4-week-old tomato plants not infected with the pathogen *B. cinerea* (-B). **A.** Expression ratios in plants inoculated with *T. harzianum* wild type strain (Th) *versus* expression levels in non-inoculated (-Th), non-infected (-B) plants. **B.** Expression levels in tomato plants inoculated with *T. harzianum* mutant Δ*epl-1* strain (*Δepl-1*) *versus* non-inoculted (-*Δepl-1*), non-infected (-B) plants. qPCR comparative calculations and representations were carried out as indicated in the legend of Supplementary Fig. 1. Numeric values are included in Supplementary Table S2b.
