# Supplementary material for: Involvement of Trichoderma harzianum Epl-1 Protein in the Regulation of Botrytis Virulence- and Tomato Defense-Related Genes
Source: Front Plant Sci. 2017 May 29;8:880. doi: 10.3389/fpls.2017.00880 (PMC5446994; doi:10.3389/fpls.2017.00880)
Supplement: Supplementary file 5 [file Image_4.PDF]

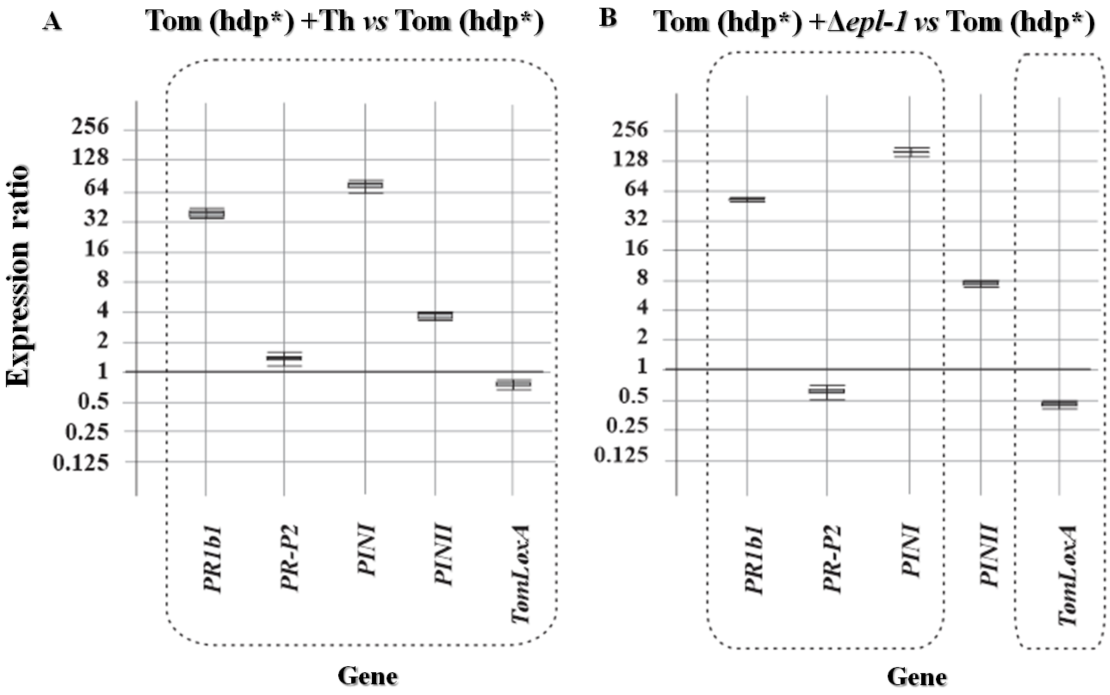

Supplementary Figure S4 - Relative expression levels of defense-related genes belonging to the SA and JA pathways in tomato hydroponic cultures [Tom (hdp*)] 24 h after *T. harzianum* strains inoculations. **A.** Expression ratio 24 h after *T. harzianum* WT strain (Th) inoculation compared with control condition [Tom (hdp*)]. **B.** Expression ratio 24 h after *T. harzianum* Δ*epl-1* strain (Δ*epl-1*) inoculation compared with control condition. qPCR comparative calculations and representations were carried out as indicated in the legend of Supplementary Fig. S1. Numeric values are included in Supplementary Table S3b.
